# Validation of an enhanced pediatric vitamin D deficiency score incorporating sun exposure timing and BMI *z*-score: analysis in a combined cohort of children

**DOI:** 10.1007/s00431-026-06890-x

**Published:** 2026-03-30

**Authors:** Valeria Calcaterra, Hellas Cena, Ginevra Biino, Ilaria Anna Maria Scavone, Alessandra Vincenti, Gianvincenzo Zuccotti

**Affiliations:** 1 Department of Internal Medicine and Therapeutics, University of Pavia, 27100 Pavia, Italy; 2 Pediatric Department, Buzzi Children’s Hospital, 20154 Milan, Italy; 3 Laboratory of Dietetics and Clinical Nutrition, Department of Public Health, Experimental and Forensic Medicine, University of Pavia, 27100 Pavia, Italy; 4 Clinical Nutrition Unit, ICS Maugeri IRCCS, 27100 Pavia, Italy; 5 Institute of Molecular Genetics, National Research Council of Italy, 27100 Pavia, Italy; 6 Department of Biomedical and Clinical Science, University of Milano, 20157 Milan, Italy

**Keywords:** Vitamin D deficiency, Sun exposure timing, BMI z-score, Questionnaire, Children, Pediatrics

## Abstract

Early identification of vitamin D (VitD) deficiency in childhood is essential to support timely prevention and intervention strategies. Building on the previously validated pediatric EVIDENCe-Q questionnaire, this study aimed to refine the tool by integrating two patient-specific weighted variables, sun exposure during peak UVB hours, and BMI *z*-score categories, both recognized determinants of circulating 25-hydroxyvitamin D (25-OH-D) levels. A total of 354 children (190F/164 M) completed a 20-item pediatric VitD risk questionnaire. Serum 25-OH-D concentrations were available for 280 participants. Three scoring algorithms were evaluated: the unweighted standard model, the sun-weighted model, and the sun + BMI-weighted model. Statistical analyses included ANOVA, Pearson correlations, and receiver operating characteristic (ROC) analyses across biochemical thresholds of vitamin D deficiency and insufficiency (< 10, < 20, and < 30 ng/mL). Vitamin D deficiency (< 10 ng/mL) was observed in 6.8% of participants, while insufficiency affected 38.1% (< 20 ng/mL) and 74.7% (< 30 ng/mL). The unweighted standard model did not discriminate among vitamin D categories (*p* = 0.622). In contrast, both weighted models showed significant discrimination (sun-weighted: *p* = 0.036; sun + BMI-weighted: *p* = 0.030). Only the sun + BMI-weighted model was significantly correlated with serum 25-OH-D levels (*r* =  − 0.13; *p* = 0.023). ROC analyses demonstrated limited accuracy for the unweighted model, whereas the weighted models showed moderate discriminatory ability, with the Sun + BMI-Weighted Model providing the strongest overall performance.

*Conclusions*: Incorporating weighted patient-specific variables improves the discriminatory capacity of the pediatric EVIDENCe-Q. Despite moderate accuracy, particularly at lower thresholds, the refined questionnaire, especially the sun + BMI-weighted version, represents a practical, noninvasive screening tool to identify children at risk of hypovitaminosis D.

**Table:** 

**What is Known:** •* Vitamin D deficiency is highly prevalent in children worldwide, even in sun-rich regions, and is influenced by factors such as sun exposure and adiposity.* • *Serum 25-OH-D measurement is the diagnostic gold standard, while questionnaire-based tools offer a non-invasive but currently less accurate alternative in pediatrics.*
**What is New:** • *Incorporating weighted factors for peak UVB sun exposure and BMI z-score signifi cantly improves the predictive performance of the pediatric EVIDENCe-Q.* • *The Sun + BMI-weighted model shows a significant correlation with serum 25-OH-D and enhances identification of children at risk of hypovitaminosis D (30 ng/mL).*

**Supplementary Information:**

The online version contains supplementary material available at 10.1007/s00431-026-06890-x.

## Introduction

Vitamin D plays a critical role in skeletal development, calcium homeostasis, and immune regulation throughout childhood [1–4]. Cutaneous synthesis induced by ultraviolet B radiation represents the primary source of vitamin D, while dietary intake from foods such as fatty fish, eggs, dairy products, and fortified items provides a smaller but relevant contribution [4–6]. Several factors, including limited sun exposure, inadequate diet, darker skin pigmentation, predominantly indoor lifestyles, and excess adiposity, increase the risk of hypovitaminosis D, a condition that in children may be associated with rickets, disturbances in mineral metabolism, and possible immunological and endocrinological consequences [7, 8].

Vitamin D deficiency is now recognized as a widespread global public health issue, significantly affecting the pediatric population. International estimates suggest that approximately 50% of children under five years of age exhibit inadequate vitamin D levels [9], while in many school-aged cohorts the combined prevalence of mild, moderate, or severe hypovitaminosis reaches 40–60% [10]. Large population-based studies report even higher rates: in certain highly urbanized regions, the combined prevalence of deficiency and insufficiency exceeds 60% [11], and in adolescent populations, deficiency rates around 50% have been documented [12]. Even in Europe and Mediterranean countries, where climatic conditions might suggest a lower risk, vitamin D insufficiency remains common. In Italy, a substantial proportion of children and adolescents present suboptimal levels, with peaks during winter months and among individuals with reduced outdoor activity or overweight/obesity [13–16].

Although serum 25-hydroxyvitamin D measurement is the gold standard for diagnosing vitamin D deficiency, routine testing in all pediatric patients is not always feasible due to logistical constraints, limited availability, or the low acceptability of blood sampling in younger children [17].

In this context, the EVIDENCe-Q project [18], developed by our research group, was established as a structured initiative aimed at creating standardized questionnaires capable of estimating the risk of vitamin D deficiency based on lifestyle, dietary, clinical, and behavioral factors, without requiring immediate blood sampling.

Building on this framework, our group focused on refining and adapting the instrument for pediatric use. The original EVIDENCe-Q questionnaire was created for adults by De Giuseppe et al. [18], offering a structured, noninvasive approach to risk stratification. It was later applied to children with obesity by Calcaterra et al. [19], demonstrating feasibility but revealing limited precision in capturing pediatric-specific determinants of vitamin D status. A more comprehensive pediatric adaptation was subsequently proposed by Calcaterra et al. [20], which improved usability but continued to underweight key predictors such as mid-day sun exposure and adiposity.

The aim of this study is to update and improve the EVIDENCe-Q pediatric questionnaire by introducing new and refined variables, and to compare the original unweighted version with two updated models incorporating weighted patient-specific factors. Specifically, we evaluated three versions of the tool: the unweighted standard model**,** the sun-weighted model, and the sun + BMI-weighted model. In developing the updated questionnaire, we incorporated two weighted variables: (1) a refined measure of sun exposure during peak UVB hours (10:00–15:00) and (2) BMI *z*-score categories, reflecting each child’s anthropometric profile and its established association with circulating 25-OH-D levels. This study assesses, within a single pediatric cohort, whether these enhancements improve the questionnaire’s sensitivity and specificity in identifying children at risk of hypovitaminosis D. The ultimate goal is to provide pediatricians with a quick, noninvasive, and reliable screening instrument to guide early decisions regarding the need for diagnostic testing or targeted nutritional and behavioral interventions.

## Patients and methods

### Study population

The combined dataset included 354 children (190 females, 164 males; mean 9.6 ± 4.5). Anthropometric characteristics did not differ significantly between males and females. Serum 25-OH-D measurements were available for 280 subjects after removal of one extreme outlier.

The protocol received approval from the Institutional Review Board of the hospital (Ethics Committee Milano Area 1; Study Registration 2020/ST/234; Protocol No. 0030785). The study was conducted in full accordance with the ethical principles of the Declaration of Helsinki and with current Italian regulations on biomedical research involving minors. Before participation, written informed consent was obtained from all parents or legal guardians after providing a comprehensive explanation of the study’s purpose, procedures, potential risks, and expected benefits. When appropriate, assent was also obtained from older children and adolescents. Confidentiality and data protection were ensured by anonymizing all clinical and questionnaire data prior to analysis.

### Anthropometry

Weight, height, body mass index (BMI), and BMI *z*-score were obtained following standardized pediatric procedures in accordance with WHO growth assessment guidelines [13, 20].

Height was measured using a wall-mounted stadiometer. Children were measured standing upright, barefoot, with heels together, knees extended, shoulders relaxed, and the head positioned in the Frankfurt horizontal plane to ensure accuracy. The back of the head, scapulae, buttocks, and heels were aligned with the vertical surface when anatomically feasible. The stadiometer’s headpiece was lowered to make firm contact with the crown of the head, and height was recorded to the nearest 0.1 cm [13, 20].

Weight was measured using a calibrated digital scale with the child wearing light clothing and no shoes. Measurements were recorded to the nearest 0.1 kg**.** Infants and toddlers unable to stand were weighed using a tared procedure or an infant digital scale [13, 20].

Anthropometric measurements were performed by trained pediatric staff to minimize interobserver variability, and instruments were regularly calibrated throughout the study period.

BMI was calculated as weight (kg)/height (m^2^). To account for age- and sex-related variability, BMI *z*-scores were derived using WHO reference standards [21]. BMI *z*-scores classify pediatric nutritional status into internationally recognized categories: ≤  − 1 SD: underweight/thinness; − 1 to + 1 SD: normal weight; and ≥  + 1 SD: overweight/obesity.

### Vitamin D assessment

Serum 25-OH-D was measured using commercial kits (Alinity i 25-OH VitD reagent kit, Libertyville Township, IL, USA) on the ARCHITECT system. Serum 25-OH-vitamin D (25-OH-D) was measured using the Alinity i system. According to Italian pediatric guidelines, severe deficiency is defined as < 10 ng/mL, deficiency as < 20 ng/mL, insufficiency as 20–29 ng/mL, and sufficiency as ≥ 30 ng/mL [22]. For the purposes of ROC analysis, we considered three cumulative reference thresholds: deficiency < 10 ng/mL; insufficiency-20 < 20 ng/mL; and insufficiency-30 < 30 ng/mL.

### Vitamin D questionnaire administration

All participants completed the pediatric adaptation of the EVIDENCe-Q questionnaire [20], previously validated in Italian pediatric populations. The instrument includes 20 items assessing dietary intake, supplementation, detailed sun-exposure habits, lifestyle behaviors including sleep patterns, skin phototype, and selected clinical risk factors. The full questionnaire is provided in the Supplementary Materials (Suppl. 1).

The questionnaire was completed by parents for younger children and self-administered by adolescents under supervision of trained clinical staff.

Sun exposure was assessed in terms of weekly frequency during peak UVB hours (10:00–15:00). The questionnaire did not quantify exact duration in minutes nor the percentage of body surface exposed. Respondents were instructed to consider habitual outdoor exposure involving face and arms without sunscreen.

#### Scoring algorithms

##### Unweighted standard model

The original scoring system of the questionnaire provided that responses assuming behavior that did not lead to a vitamin D deficiency were assigned a score of zero, while those potentially leading to deficiency were assigned a score of 1 (if the response mode was dichotomous) or greater than 1, increasing by one unit for each answer that assumed a progressively worse behavior. Consistent with this rationale, we retained an additive model also for the new scoring variants we want to test. The original scoring assigns non-weighted integer values to questionnaire responses, yielding a global score reflecting cumulative risk of vitamin D deficiency. Scores typically range between 10 and 34 points.

##### Sun-weighted model

Based on preliminary findings from the 154-subject subset and considerations about UVB effectiveness, we revised the scoring of sun-exposure frequency:

- Sun exposure between 10:00 and 15:00:
  - 7–6 days/week =  − 3 points
  - 5–3 days/week =  − 2 points
  - 2–1 days/week =  − 1 point
  - 0 days/week = 0
- Exposure outside 10:00–15:00 → 0 points

This modification introduces a protective negative weight for midday sun exposure, which was not represented in the original tool where all sun exposure categories were scored identically (0 points).

##### Sun + BMI-weighted model

Given the well-documented association between higher adiposity and lower vitamin D levels, BMI *z*-score categories were integrated:

- BMI z-score –1 to + 1 = 0 points (normal weight)
- BMI z-score –1 to –2 or + 1 to + 2 =  + 1 point (underweight or overweight)
- BMI z-score ≤  − 2 or ≥  + 2 =  + 2 points (thinness or obesity)

The sun + BMI-weighted model therefore incorporates the sun-weighted model plus anthropometric weighting, producing a total range of approximately 5–28 points.

### Statistical analysis

Anthropometric variables and questionnaire scores were summarized as means ± standard deviations (SD), medians, and ranges. Sex differences were assessed using *t*-tests or Mann–Whitney *U* tests.

To determine whether questionnaire scores differed among subjects classified as deficient, insufficient, or sufficient, we performed one-way ANOVA. Post-hoc multiple comparisons were not required, as our primary goal was to assess whether global differences existed among categories.

Correlation between questionnaire scores and serum 25-OH-D was examined using Pearson correlation coefficients. The goal was to determine whether higher questionnaire scores corresponded to lower biochemical vitamin D concentrations.

Receiver operating characteristic (ROC) curves were generated to evaluate the ability of each scoring system to distinguish: vitamin D deficiency (< 10 ng/mL); insufficiency < 20 ng/mL; and insufficiency < 30 ng/mL.

For each ROC curve, we calculated: AUC (area under the curve); standard error (SE); optimal cut-off point identified using the Youden index; sensitivity, specificity, and predictive values.

After determining optimal cut-offs, we applied these thresholds to the full cohort to estimate the questionnaire-based prevalence of deficiency and insufficiency, comparing those estimates with actual biochemical prevalence.

Data were managed and analyzed using STATA software version 18 (College Station, TX, USA).

## Results

### Participant characteristics

Clinical and anthropometric characteristics of the sample are reported in Table 1. No significant sex differences were observed for age, weight, height, BMI, or BMI z-score. Among the 354 participants, 281 had available serum 25-OH-vitamin D measurements; after exclusion of one outlier, 280 subjects were included in the analysis. Mean serum 25-OH-D concentration was 23.6 ± 9.9 ng/mL, with no significant difference between males and females. Vitamin D status distribution was as follows: 6.76% were deficient (< 10 ng/mL), 31.32% insufficient (< 20 ng/mL), and 67.97% insufficient when applying the < 30 ng/mL threshold.

**Table 1:** Description of anthropometric data

	Females, ***n*** = 190			Males, ***n*** = 164		
	Mean (SD)	Median	Min–max	Mean (SD)	Median	min–max
Age, y	9.44 (4.48)	9.82	1–17.9	9.73 (4.46)	10.3	0.5–17.9
Weight, kg	39.36 (22.77)	36.2	7.5–105	42.43 (25.94)	35.65	6.1–115
Height, cm	57.08 (69.35)	1.58	0.7–180	68.59 (71.89)	1.78	0.7–184
BMI, kg/m^2^	20.55 (6.45)	18.82	9.5–47.4	20.52 (6.27)	18.54	11.3–37
BMI zscore	0.71 (1.74)	0.64	− 6.8–4.1	0.71 (1.81)	0.71	− 4.6–4.1

*BMI* body mass index, *SD* standard deviation

### Questionnaire score distributions

**Fig. 1:**
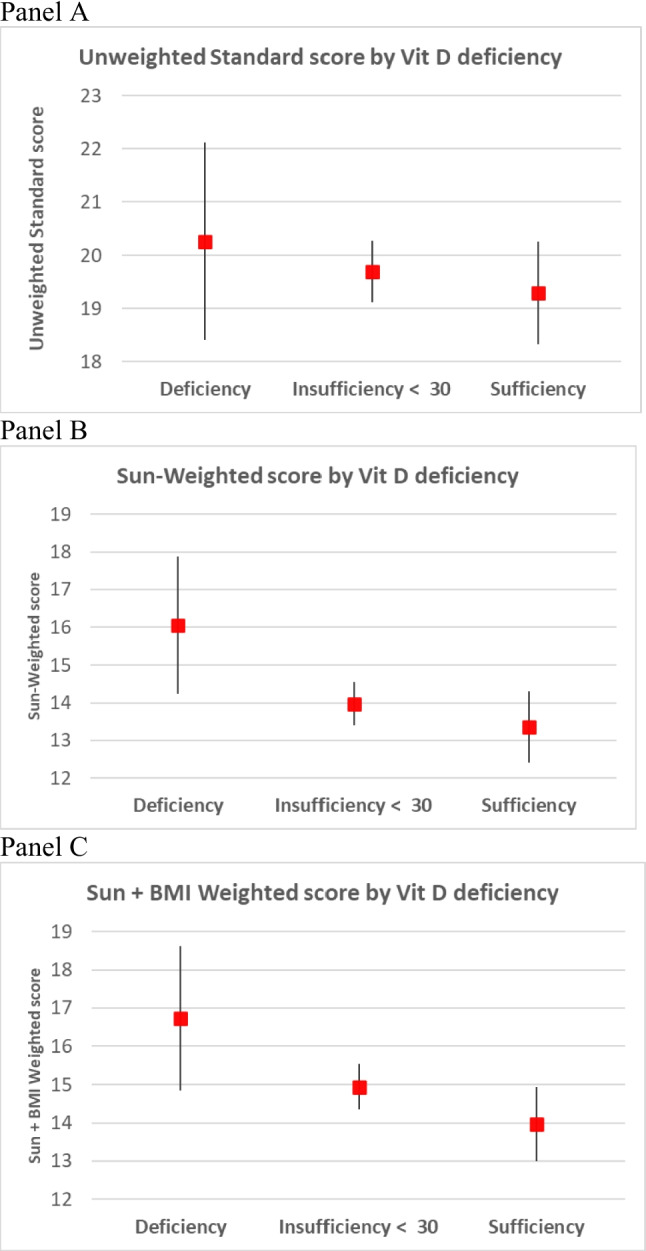
Mean questionnaire scores (± 95% confidence intervals) across vitamin D status categories (deficiency, insufficiency < 30 ng/mL, sufficiency) for the three scoring algorithms: **A** Unweighted standard model (ANOVA *p* = 0.622), **B** sun-weighted model (ANOVA *p* = 0.036), and (**C**) sun–BMI-weighted model (ANOVA *p* = 0.030)

The three questionnaire-derived scores (unweighted standard model**,** sun-weighted model, and sun + BMI-weighted model) did not differ significantly between males and females (19.6 ± 4.1, 13.9 ± 4.0, and 14.8 ± 4.2, respectively). The unweighted standard model ranged from 11 to 34, whereas both modified versions ranged from 5 to 28. Higher mean scores were observed among participants classified as vitamin D deficient, particularly for the modified and BMI-based scores (Table 2). Analysis of variance confirmed significant differences of mean score values across vitamin D categories for the sun-weighted model (*p* = 0.036) and the sun + BMI-weighted model (*p* = 0.030), but not for the unweighted standard model (Fig. 1A–C), indicating that the refined scoring systems provide improved sensitivity to differences in vitamin D status compared with the original questionnaire formulation.

**Table 2:** Summary of ROC analysis results using the 3 reference variables

	Deficiency	Insuff-20	Insuff-30
Optimal operating slope:	1	1	1
Optimal cut-off:	25	24	24
Optimal sensitivity:	31.58%	25.23%	19.05%
Optimal specificity:	87.79%	88.51%	90.14%
Clinical information statistic:	0.194	0.137	0.092
Area under the ROC Curve:	0.599	0.590	0.562
SE of Area (Hanley):	0.079	0.036	0.039
Sample size:	281	281	281

*Insuff-20* insufficiency < 20 ng/mL, *Insuff-30* insufficiency < 30 ng/mL

### Correlation between questionnaire scores and serum 25-OH-D

All three scores showed weak negative linear correlations with serum vitamin D levels: *r* =  − 0.10 (*p* = 0.097) for the unweighted standard model, *r* =  − 0.11 (*p* = 0.072) for the sun-weighted model, and *r* =  − 0.13 (*p* = 0.023) for the sun + BMI weighted model, the latter being the only statistically significant correlation. Incorporating the BMI z-score improved the questionnaire’s discriminatory capacity for detecting individuals at risk of low vitamin D status.

### ROC analysis

#### ROC analysis of the unweighted standard model

The ROC analysis using the unweighted standard model showed that the optimal cut-off for detecting biochemical vitamin D deficiency (< 10 ng/mL) was 25, corresponding to an AUC of 0.599. At this threshold, sensitivity was 31.6% and specificity was 87.7%, indicating that the unweighted standard model performed relatively better in identifying true deficiency, although its overall performance declined progressively at higher vitamin D thresholds.

For insufficiency defined as < 20 ng/mL, the optimal cut-off was 24, yielding an AUC of 0.590. While sensitivity remained high (88.5%), specificity decreased markedly (25.2%), resulting in reduced discriminative ability. When insufficiency was defined as < 30 ng/mL, the optimal cut-off remained 24, with an AUC of 0.562; sensitivity was 90.1% and specificity was 19.0%.

The diagnostic performance of the Unweighted Standard Model across the three vitamin D cut-off thresholds is reported in Supplementary Materials (Table S1 and Figure S1, Panel 1).

The summary of ROC analysis results using the 3 reference variables is reported in Table 3.

**Table 3:** Comparison of vitamin D deficiency and insufficiency prevalence obtained using biochemical thresholds and questionnaire-derived cut-offs

	Unweighted standard model cut-offs		Sun-weighted model cut-offs		Sun-weighted model cut-offs	
	(25-OH-D3) *n* (%)	Score cut-off on total sample *n* (%)	(25-OH-D3) n (%)	Score cut-off on total sample *n* (%)	(25-OH-D3) *n* (%)	Score cut-off on total sample *n* (%)
Deficiency < 10 ng/mL	19 (6.8)	51 (14.4)	19 (6.8)	123 (34.7)	19 (6.8)	218 (61.6)
Insufficiency < 20 ng/mL	107 (38.1)	64 (18.1)	107 (38.1)	312 (88.1)	107 (38.1)	279 (78.8)
Insufficiency < 30 ng/mL	210 (74.7)	64 (18.1)	210 (74.7)	280 (79.1)	210 (74.7)	279 (78.8)

Comparison of vitamin D deficiency and insufficiency prevalence obtained using biochemical thresholds and questionnaire-derived cut-offs is shown in Table 4.

**Table 4:** Summary of ROC analysis results for sun-weighted model using the 3 reference variables

	Deficiency	Insuff-20	Insuff-30
Optimal operating slope:	1	1	1
Optimal cut-off:	16	10	11
Optimal sensitivity:	68.42%	94.40%	84.76%
Optimal specificity:	68.70%	16.67%	36.72%
Clinical information statistic:	0.371	0.111	0.214
Area under the ROC curve:	0.719	0.569	0.623
SE of area (Hanley):	0.061	0.034	0.044
Sample size:	281	281	281

*Insuff-20* insufficiency < 20 ng/mL, *Insuff-30* insufficiency < 30 ng/mL

The table shows that the unweighted standard model tends to classify a higher number of children as potentially deficient or insufficient when stricter thresholds are applied, while it becomes more accurate when using a broader cut-off such as 30 ng/mL.

#### ROC analysis of the sun-weighted model

Repeating the ROC analysis using the sun-weighted model (excluding BMI) showed an optimal cut-off of 16 for detecting deficiency (< 10 ng/mL), corresponding to an AUC of 0.719. For insufficiency defined as < 20 ng/mL, the optimal threshold was 10, with an AUC of 0.569, whereas for insufficiency < 30 ng/mL the best cut-off was 11, with an AUC of 0.623 (Supplementary Materials, Table S2 and Figure S1, Panel 2).

A detailed summary of the sun-weighted model performance is provided in Tables 5

**Table 5:** Summary of ROC analysis results for sun + BMI-weighted model using the 3 reference variables

	Deficiency	Insuff-20	Insuff-30
Optimal operating slope:	1	1	1
Optimal cut-off:	14	12	12
Optimal sensitivity:	89–47%	86.92%	84.29%
Optimal specificity:	41.76%	27.01%	39.44%
Clinical information statistic:	0.312	0.139	0.237
Area under the ROC Curve:	0.691	0.587	0.627
SE of area (Hanley):	0.063	0.034	0.043
Sample size:	280	281	281

*Insuff-20* insufficiency < 20 ng/mL, *Insuff-30* insufficiency < 30 ng/mL

For the sun-weighted model, Table 4 shows that this version also overestimates deficiency and insufficiency at stricter thresholds, although to a lesser extent than the unweighted standard model. The discrepancy remains evident for < 10 ng/mL and < 20 ng/mL, where the score-based prevalence is still considerably higher than the biochemical values. However, as with the unweighted standard model, the sun-weighted version becomes more accurate when applying the broader < 30 ng/mL threshold, where questionnaire-derived and biochemical prevalences align more closely. Compared with the unweighted model, the sun-weighted model provides a slightly more balanced classification across categories, but still tends to inflate estimates at the lower cut-off levels.

#### ROC analysis of the sun + BMI-weighted model

Updated ROC analyses identified new optimal thresholds for the sun + BMI-weighted model: a cut-off of 14 for detecting deficiency (< 10 ng/mL), corresponding to an AUC of 0.691; a cut-off of 12 for insufficiency < 20 ng/mL, with an AUC of 0.587; and the same cut-off of 12 for insufficiency < 30 ng/mL, with an AUC of 0.627 (Supplementary Materials Table S3 and Figure S1, Panel 3).

Overall, performance of sun + BMI-weighted model was highest for identifying biochemical deficiency; summary metrics are reported in Table 6.

**Table 6:** Mean values and SD of questionnaire scores by Vit D status

		Unweighted standard model	Sun-weighted model	Sun + BMI weighted model
	***n***	Mean (SD)	Mean (SD)	Mean (SD)
Deficiency	19	20.3 (5.26)	16 (4.17)	16.7 (4.46)
Insufficiency 30	191	19.7 (4.1)	14 (3.74)	14.9 (3.88)
Sufficiency	70	19.3 (3.78)	13.4 (4.69)	14 (4.81)

*BMI* body mass index, *SD* standard deviation

In Table 4, the sun + BMI-weighted model identifies more children as potentially deficient or insufficient at the stricter thresholds compared with the sun-weighted and unweighted models. Although this leads to greater overestimation, it also increases sensitivity and reduces the likelihood of missing children at risk. At the broader < 30 ng/mL threshold, its estimates are similar to those obtained with the other models and align more closely with biochemical data.

#### Overall diagnostic performance of the scoring systems

Across all analyses, ROC curves displayed moderate discriminatory ability, indicating acceptable accuracy for identifying low vitamin D status. Although both refined scoring systems improved upon the original questionnaire, they tended to overestimate true biochemical deficiency. Performance was more reliable for detecting insufficiency defined as 25-OH-D < 30 ng/mL.

## Discussion

This study assessed the ability of three questionnaire-derived scoring models, the unweighted standard model (used as the reference), the sun-weighted model, and the sun + BMI-weighted model, to identify vitamin D deficiency and insufficiency in a pediatric population. The results indicate that both refined weighted models outperform the standard unweighted questionnaire in discriminating vitamin D status, although their accuracy varies according to the biochemical thresholds applied. Overall, the questionnaire demonstrated moderate discriminatory capacity with clinically meaningful sensitivity, particularly at broader (< 30 ng/mL) thresholds.

These observations align with previous research which has demonstrated that simple questionnaire-based screening tools may serve as practical first-line instruments for vitamin D risk stratification, [23, 24] particularly when biochemical testing is impractical or resource-limited. For instance, the adult-focused EVIDENCe–Q [18], originally developed for vitamin D deficiency screening, has been adapted and tested in pediatric populations with acceptable performance, showing that the questionnaire is useful for identifying children at risk of vitamin D deficiency or insufficiency [25]. Similarly, in a population of children with obesity, a modified questionnaire demonstrated inverse correlations between adiposity indices and serum vitamin D, and proposed cut-offs for deficiency and insufficiency, further underscoring the relevance of including BMI in risk assessments [19].

Nonetheless, even though modified versions of the questionnaire have improved performance compared with the original standard format, their accuracy remains limited. The modest AUC values and low specificity are similar to those reported in other validation studies, which consistently classify these tools as useful for screening but insufficient for diagnostic purposes [19, 25]. This limitation is inherent to instruments based on self-reported behaviors or risk factors rather than biochemical measurements: recall bias, differences in physical activity level and other unmeasured factors contribute to residual variability in serum 25-OH-D that a questionnaire alone cannot adequately capture.

The revised scoring system was not derived through automated predictive modeling but rather through physiologically informed pragmatic weighting of well-established determinants of vitamin D status. As such, it should be interpreted as a clinically guided additive risk estimation tool rather than a predictive algorithm.

Our finding that only the BMI-based score showed a significant correlation with serum 25-OH-D matches the biological and epidemiological evidence that higher adiposity is associated with lower circulating vitamin D levels, likely due to volumetric dilution and sequestration in adipose tissue [26–28]. Indeed, recent population-based data confirm that overweight/obesity and reduced outdoor activity are significant predictors of vitamin D insufficiency in children and adolescents [29].

The discrepancy between ROC-derived cut-offs and real-world biochemical prevalences, particularly the overestimation of deficiency, further underscores the need for caution when interpreting high questionnaire scores. This pattern is consistent with previous findings [19, 25]: questionnaires typically prioritize sensitivity to avoid missing deficient individuals [30], but this comes at the expense of specificity, increasing false positives. Nonetheless, the good agreement between questionnaire-based and biochemical prevalences for insufficiency < 30 ng/mL indicates a valuable strength: the instrument appears well suited for detecting broader hypovitaminosis D in population-level surveillance, supporting early lifestyle interventions and guiding targeted confirmatory testing.

From a public health and clinical standpoint, these findings highlight the valuable potential of refined questionnaire-based tools for screening vitamin D insufficiency in children. In contexts where routine biochemical testing is impractical, expensive, or logistically challenging, such questionnaires can serve as an efficient first step, helping clinicians prioritize which individuals may benefit most from further evaluation. In routine pediatric practice, the questionnaire may serve as a first-line triage instrument to identify children in whom serum 25-OH-D testing is most appropriate, rather than as a standalone diagnostic instrument.

At the same time, it remains important to acknowledge that these tools cannot substitute for serum 25-OH-D measurement when precise diagnostic accuracy is required, for example before initiating supplementation, evaluating fracture risk, or monitoring treatment response. This consideration aligns with recent consensus statements, which continue to recommend biochemical confirmation for diagnosis and therapeutic decisions [31, 32], while also recognizing that assessing behavioral and environmental risk factors plays a meaningful role in identifying children who may be at increased risk [33–35].

The strengths of this study include the relatively large pediatric sample, the availability of both anthropometric and biochemical data, and the systematic comparison of multiple scoring algorithms, which allowed direct evaluation of the added value of incorporating behavioral and body composition variables. Limitations include the cross-sectional design, the reliance on self-reported sun exposure (with potential recall bias), and the lack of detailed information on factors such as physical activity levels which may influence vitamin D status but were not captured with sufficient granularity. In addition, the relatively low number of children with severe vitamin D deficiency may have limited the ability of the models to accurately discriminate at lower serum cut-off levels.

## Conclusions

The sun-weighted and sun + BMI-weighted models demonstrated superior discriminatory capacity compared with the unweighted standard model, although all three tools showed only moderate accuracy. The refined scores improved the identification of biochemical deficiency and, more consistently, of vitamin D insufficiency, with their most reliable performance observed at higher serum thresholds (< 30 ng/mL)**.** While all models tended to overestimate deficiency and insufficiency at lower cut-off levels, their estimates aligned more closely with biochemical prevalence when more inclusive thresholds were applied.

Despite limited specificity, these updated scoring systems represent a practical, non-invasive first-line strategy for identifying children at risk of hypovitaminosis D and for prioritizing those who may require confirmatory biochemical testing. Further refinement of the questionnaire, incorporating additional clinically relevant behavioral and environmental factors, could certainly enhance its predictive performance in future applications.

## Supplementary Information

Below is the link to the electronic supplementary material.

ESM 1 (DOCX 169 KB)

## Data Availability

All data supporting the findings of this study are available within the paper and its Supplementary Information.
