# Supplementary material for: Validation of an enhanced pediatric vitamin D deficiency score incorporating sun exposure timing and BMI z-score: analysis in a combined cohort of children
Source: Eur J Pediatr. 2026 Mar 30;185(4):226. doi: 10.1007/s00431-026-06890-x (PMC13035619; doi:10.1007/s00431-026-06890-x)
Supplement: Supplementary file 1 — (DOCX 169 KB) [file 431_2026_6890_MOESM1_ESM.docx]

**SUPPLEMENTARY**

**TABLES**

#### **Table S1**. Diagnostic performance of the Unweighted Standard Model across three vitamin D cut-off thresholds: A) Deficiency (<10 ng/mL); B) Insufficiency Shortage-20 (<20 ng/mL); C) Insufficiency Shortage-30 (<30 ng/mL)

| **A) Deficiency -10 prevalence** | | | | | | |
| --- | --- | --- | --- | --- | --- | --- |
| Score Cut-off | Sensitivity | 95% CI | Specificity | 95% CI | # Pos | # Neg |
| . | 0.00% | (0 - 0.209) | 100.00% | (0.982 - 1) |  |  |
| 34 | 0.00% | (0 - 0.209) | 99.62% | (0.976 - 1) | 0 | 1 |
| 30 | 5.26% | (0.003 - 0.281) | 98.86% | (0.964 - 0.997) | 1 | 2 |
| 29 | 10.53% | (0.018 - 0.345) | 96.95% | (0.938 - 0.986) | 1 | 5 |
| 25^a^ | 31.58% | (0.136 - 0.565) | 87.79% | (0.831 - 0.914) | 4 | 24 |
| 16 | 89.47% | (0.655 - 0.982) | 13.74% | (0.099 - 0.186) | 11 | 194 |
| 11 | 100.00% | (0.791 - 1) | 0.00% | (0 - 0.018) | 2 | 36 |
| Deficiency prevalence (score≥25)=14.4%; ROC curve AUC= 0.599; SE= 0.079; #Pos = positive predicted value; #Neg = negative predicted value; ^a^ Best cut-off for standard unweighted score predicting vit D deficiency | | | | | | |
| **B) Insufficiency Shortage-20 prevalence** | | | | | | |
| Score Cut-off | Sensitivity | 95% CI | Specificity | 95% CI | # Pos | # Neg |
| . | 0.00% | (0 - 0.043) | 100.00% | (0.973 - 1) |  |  |
| 34 | 0.94% | (0 - 0.058) | 100.00% | (0.973 - 1) |  |  |
| 30 | 2.80% | (0.007 - 0.086) | 99.43% | (0.964 - 1) | 3 | 1 |
| 25 | 20.56% | (0.136 - 0.297) | 90.81% | (0.852 - 0.945) | 19 | 15 |
| 24^a^ | 25.23% | (0.176 - 0.347) | 88.51% | (0.826 - 0.927) | 5 | 4 |
| 20 | 55.14% | (0.452 - 0.647) | 56.32% | (0.486 - 0.638) | 32 | 56 |
| 15 | 94.39% | (0.877 - 0.977) | 10.35% | (0.064 - 0.161) | 42 | 80 |
| 14 | 97.20% | (0.914 - 0.993) | 6.90% | (0.038 - 0.12) | 3 | 6 |
| 11 | 100.00% | (0.957 - 1) | 0.00% | (0 - 0.027) | 3 | 12 |
| Shortage-20 prevalence (score≥24)= 18.1%; ROC curve AUC= 0.590; SE= 0.036; #Pos = positive predicted value; #Neg = negative predicted value; ^a^ Best cut-off for standard unweighted score predicting vit D shortage-20 | | | | | | |
| **C) Insufficiency Shortage-30 prevalence** | | | | | | |
| Score Cut-off | Sensitivity | 95% CI | Specificity | 95% CI | # Pos | # Neg |
| . | 0.00% | (0 - 0.022) | 100.00% | (0.936 - 1) |  |  |
| 30 | 1.91% | (0.006 - 0.051) | 100.00% | (0.936 - 1) |  |  |
| 25 | 15.71% | (0.112 - 0.215) | 92.96% | (0.837 - 0.974) | 33 | 5 |
| 24^a^ | 19.05% | (0.141 - 0.252) | 90.14% | (0.802 - 0.956) | 7 | 2 |
| 14 | 95.71% | (0.918 - 0.979) | 8.45% | (0.035 - 0.181) | 161 | 58 |
| 11 | 100.00% | (0.978 - 1) | 0.00% | (0 - 0.064) | 9 | 6 |
| Shortage-30 prevalence (score≥24)=18.1%; ROC curve AUC= 0.562; SE=0.039 ; #Pos = positive predicted value; #Neg = negative predicted value; ^a^ Best cut-off for standard unweighted score predicting vit D shortage-30 | | | | | | |

#### **Table S2** Diagnostic performance of the EVIDENCe-Q Sun-Weighted Model across three vitamin D cut-off thresholds: A) Deficiency (<10 ng/mL); B) Insufficiency Shortage-20 (<20 ng/mL); C) Insufficiency Shortage-30 (<30 ng/mL)

| **A) Deficiency -10 prevalence** | | | | | | |
| --- | --- | --- | --- | --- | --- | --- |
| Score Cut-off | Sensitivity | 95% CI | Specificity | 95% CI | # Pos | # Neg |
| . | 0.00% | (0 - 0.209) | 100.00% | (0.982 - 1) |  |  |
| 28 | 0.00% | (0 - 0.209) | 99.62% | (0.976 - 1) | 0 | 1 |
| 23 | 5.26% | (0.003 - 0.281) | 97.71% | (0.948 - 0.991) | 1 | 5 |
| 16^a^ | 68.42% | (0.435 - 0.864) | 68.70% | (0.627 - 0.742) | 12 | 76 |
| 14 | 84.21% | (0.595 - 0.958) | 52.67% | (0.464 - 0.588) | 3 | 42 |
| 13 | 89.47% | (0.655 - 0.982) | 40.46% | (0.345 - 0.467) | 1 | 32 |
| 6 | 100.00% | (0.791 - 1) | 0.38% | (0 - 0.024) |  |  |
| 5 | 100.00% | (0.791 - 1) | 0.00% | (0 - 0.018) | 2 | 106 |
| Deficiency prevalence (score≥16)=34.7%; ROC curve AUC= 0.719; SE= 0.061; #Pos = positive predicted value; #Neg = negative predicted value; ^a^ Best cut-off for Sun-Weighted Model predicting vit D deficienc*y* | | | | | | |
| **B) Insufficiency Shortage-20 prevalence** | | | | | | |
| Score Cut-off | Sensitivity | 95% CI | Specificity | 95% CI | # Pos | # Neg |
| . | 0.00% | (0 - 0.043) | 100.00% | (0.973 - 1) |  |  |
| 25 | 1.87% | (0.003 - 0.073) | 100.00% | (0.973 - 1) |  |  |
| 12 | 78.51% | (0.693 - 0.856) | 32.18% | (0.254 - 0.397) | 84 | 118 |
| 10^a^ | 94.40% | (0.877 - 0.977) | 16.67% | (0.116 - 0.232) | 17 | 27 |
| 6 | 100.00% | (0.957 - 1) | 0.58% | (0 - 0.036) |  |  |
| 5 | 100.00% | (0.957 - 1) | 0.00% | (0 - 0.027) | 6 | 29 |
| Shortage-20 prevalence (score≥10)= 88.1%; ROC curve AUC= 0.569; SE= 0.034; #Pos = positive predicted value; #Neg = negative predicted value; ^a^ Best cut-off for Sun-Weighted Model predicting vit D shortage-20 | | | | | | |
| **C) Insufficiency Shortage-30 prevalence** | | | | | | |
| Score Cut-off | Sensitivity | 95% CI | Specificity | 95% CI | # Pos | # Neg |
| . | 0.00% | (0 - 0.022) | 100.00% | (0.936 - 1) |  |  |
| 25 | 0.95% | (0.002 - 0.038) | 100.00% | (0.936 - 1) |  |  |
| 12 | 77.14% | (0.708 - 0.825) | 43.66% | (0.321 - 0.559) | 162 | 40 |
| 11^a^ | 84.76% | (0.79 - 0.892) | 36.62% | (0.257 - 0.49) | 16 | 5 |
| 10 | 92.38% | (0.877 - 0.954) | 26.76% | (0.173 - 0.388) | 16 | 7 |
| 7 | 99.05% | (0.962 - 0.998) | 5.63% | (0.018 - 0.145) | 14 | 15 |
| 6 | 100.00% | (0.978 - 1) | 1.41% | (0.001 - 0.087) |  |  |
| 5 | 100.00% | (0.978 - 1) | 0.00% | (0 - 0.064) | 2 | 4 |
| Shortage-30 prevalence (score≥11)=79.1%; ROC curve AUC= 0.623; SE=0.044; #Pos = positive predicted value; #Neg = negative predicted value; ^a^ Best cut-off for Sun-Weighted Model predicting vit D shortage-30 | | | | | | |

#### **Table S3**. Diagnostic performance of the EVIDENCe-Q Sun+BMI Weighted Model across three vitamin D cut-off thresholds: A) Deficiency (<10 ng/mL); B) Insufficiency Shortage-20 (<20 ng/mL); C) Insufficiency Shortage-30 (<30 ng/mL)

| **A) Deficiency -10 prevalence** | | | | | | |
| --- | --- | --- | --- | --- | --- | --- |
| Score Cut-off | Sensitivity | 95% CI | Specificity | 95% CI | # Pos | # Neg |
| . | 0.00% | (0 - 0.209) | 100.00% | (0.982 - 1) |  |  |
| 28 | 0.00% | (0 - 0.209) | 99.62% | (0.975 - 1) | 0 | 1 |
| 25 | 5.26% | (0.003 - 0.281) | 98.85% | (0.964 - 0.997) | 1 | 2 |
| 24 | 10.53% | (0.018 - 0.345) | 97.32% | (0.943 - 0.988) | 1 | 4 |
| 17 | 52.63% | (0.295 - 0.748) | 70.88% | (0.649 - 0.762) | 8 | 69 |
| 16 | 68.42% | (0.435 - 0.864) | 60.15% | (0.539 - 0.661) | 3 | 28 |
| 15 | 78.95% | (0.539 - 0.93) | 50.96% | (0.447 - 0.572) | 2 | 24 |
| **14^a^** | 89.47% | (0.655 - 0.982) | 41.76% | (0.358 - 0.48) | 2 | 24 |
| 6 | 100.00% | (0.791 - 1) | 0.00% | (0 - 0.018) | 2 | 109 |
| Deficiency prevalence (score≥14)=61.6%; ROC curve AUC= 0.691; SE= 0.063; #Pos = positive predicted value; #Neg = negative predicted value; ^a^ Best cut-off for score BMI predicting vit D deficiency | | | | | | |
| **B) Insufficiency Shortage-20 prevalence** | | | | | | |
| Score Cut-off | Sensitivity | 95% CI | Specificity | 95% CI | # Pos | # Neg |
| . | 0.00% | (0 - 0.043) | 100.00% | (0.973 - 1) |  |  |
| 28 | 0.94% | (0 - 0.058) | 100.00% | (0.973 - 1) |  |  |
| 25 | 2.80% | (0.007 - 0.086) | 99.43% | (0.964 - 1) | 3 | 1 |
| **12^a^** | 86.92% | (0.787 - 0.924) | 27.01% | (0.207 - 0.344) | 90 | 126 |
| 11 | 92.52% | (0.854 - 0.965) | 20.69% | (0.151 - 0.276) | 6 | 11 |
| 8 | 99.07% | (0.942 - 1) | 4.60% | (0.022 - 0.092) | 7 | 28 |
| 6 | 100.00% | (0.957 - 1) | 0.00% | (0 - 0.027) | 1 | 8 |
| Shortage-20 prevalence (score≥12)= 78.8%; ROC curve AUC= 0.587; SE= 0. 034; #Pos = positive predicted value; #Neg = negative predicted value; ^a^ Best cut-off for score BMI predicting vit D shortage-20 | | | | | | |
| **C) Insufficiency Shortage-30 prevalence** | | | | | | |
| Score Cut-off | Sensitivity | 95% CI | Specificity | 95% CI | # Pos | # Neg |
| . | 0.00% | (0 - 0.022) | 100.00% | (0.936 - 1) |  |  |
| 28 | 0.48% | (0 - 0.03) | 100.00% | (0.936 - 1) |  |  |
| 12^a^ | 84.29% | (0.785 - 0.888) | 39.44% | (0.283 - 0.518) | 177 | 43 |
| 11 | 90.00% | (0.849 - 0.936) | 32.39% | (0.22 - 0.447) | 12 | 5 |
| 10 | 93.81% | (0.894 - 0.965) | 21.13% | (0.127 - 0.327) | 8 | 8 |
| 7 | 99.05% | (0.962 - 0.998) | 4.23% | (0.011 - 0.127) | 11 | 12 |
| 6 | 100.00% | (0.978 - 1) | 0.00% | (0 - 0.064) | 2 | 3 |
| Shortage-30 prevalence (score≥12)=78.8%; ROC curve AUC= 0.627; SE=0. 043; ^a^ Best cut-off for score BMI predicting vit D shortage-30;*#Pos = positive predicted value; #Neg = negative predicted value* | | | | | | |

**FIGURE**

**Figure S1.** Receiver operating characteristic (ROC) curves illustrating the discriminatory performance of the three questionnaire scoring models for identifying vitamin D deficiency (<10 ng/mL; A), insufficiency <20 ng/mL (B), and insufficiency <30 ng/mL (C). Panels show, respectively, the Unweighted Standard Model (Panel 1), the Sun-Weighted Model (Panel 2), and the Sun+BMI-Weighted Model (Panel 3).

| Panel 1 | | |
| --- | --- | --- |
| A | B | C |
|  |  |  |
| Panel 2 | | |
| A | B | C |
|  |  |  |
| Panel 3 | | |
| A | B | C |
|  |  |  |
